# A New Picture of the Global Impacts of El Nino-Southern Oscillation

**DOI:** 10.1038/s41598-019-54090-5

**Published:** 2019-11-26

**Authors:** Jialin Lin, Taotao Qian

**Affiliations:** Atmospheric Science Program, The Ohio State University, Columbus, Ohio, USA

**Keywords:** Atmospheric dynamics, Natural hazards, Physical oceanography

## Abstract

The El Nino-Southern Oscillation (ENSO) is the dominant interannual variability of Earth’s climate system and plays a central role in global climate prediction. Outlooks of ENSO and its impacts often follow a two-tier approach: predicting ENSO sea surface temperature anomaly in tropical Pacific and then predicting its global impacts. However, the current picture of ENSO global impacts widely used by forecasting centers and atmospheric science textbooks came from two earliest surface station datasets complied 30 years ago, and focused on the extreme phases rather than the whole ENSO lifecycle. Here, we demonstrate a new picture of the global impacts of ENSO throughout its whole lifecycle based on the rich latest satellite, *in situ* and reanalysis datasets. ENSO impacts are much wider than previously thought. There are significant impacts unknown in the previous picture over Europe, Africa, Asia and North America. The so-called “neutral years” are not neutral, but are associated with strong sea surface temperature anomalies in global oceans outside the tropical Pacific, and significant anomalies of land surface air temperature and precipitation over all the continents.

## Introduction

The dominant interannual variability of global climate system is ENSO^1–7^, which plays a central role in seasonal to decadal global climate prediction^8–15^. Currently, outlooks of ENSO and its impacts generally follow a two-tier approach. The first step is to predict ENSO sea surface temperature (SST) anomaly in tropical Pacific Ocean, and the second step is to predict the global impacts of ENSO SST anomaly. The second step is the focus of this study.

The widely-used schematic of global impacts of ENSO is from Trenberth *et al*.^8^. It is being used by major international and national climate prediction centers such as National Centers for Environmental Prediction (NCEP) Climate Prediction Center (CPC)^9^, International Research Institute (IRI)^10^, NOAA Pacific Marine Environmental Laboratory (PMEL)^11^, and UK Met Office^12^. It is also being used by many public education websites such as the World Meteorological Organization (WMO) ENSO website^13^, U.S. Government’s Global.gov^14^, NOAA’s Climate.gov^15^, as well as most, if not all, atmospheric science textbooks.

However, the schematic of Trenberth *et al*.^8^ is a summary of the pioneering works of Ropelewski and Halpert (1986^16^, 1987^17^, 1989^18^) and Halpert and Ropelewski (1992)^19^, which were based a single dataset – the World Monthly Surface Station Climatology (WMSSC) dataset compiled by National Center for Atmospheric Research (NCAR) in the 1980s, supplemented by Aceituno^20^ for South America stations. In the past 30 years, many new surface station datasets have been collected, many satellite measurements have been made, many new techniques have been developed for constructing global analysis, and many high-quality global reanalyses became available. In addition, the current picture focused on extreme phases of ENSO (El Nino and La Nina) rather than the whole ENSO lifecycle^8,21,22^. Therefore, time is ripe to revisit the picture of global impacts of ENSO lifecycle.

Here, we demonstrate a new picture of the global impacts of ENSO throughout its whole lifecycle based on the rich latest satellite, *in situ* and reanalysis datasets. See Methods section for details about the datasets and analysis methods.

**Figure 1:**
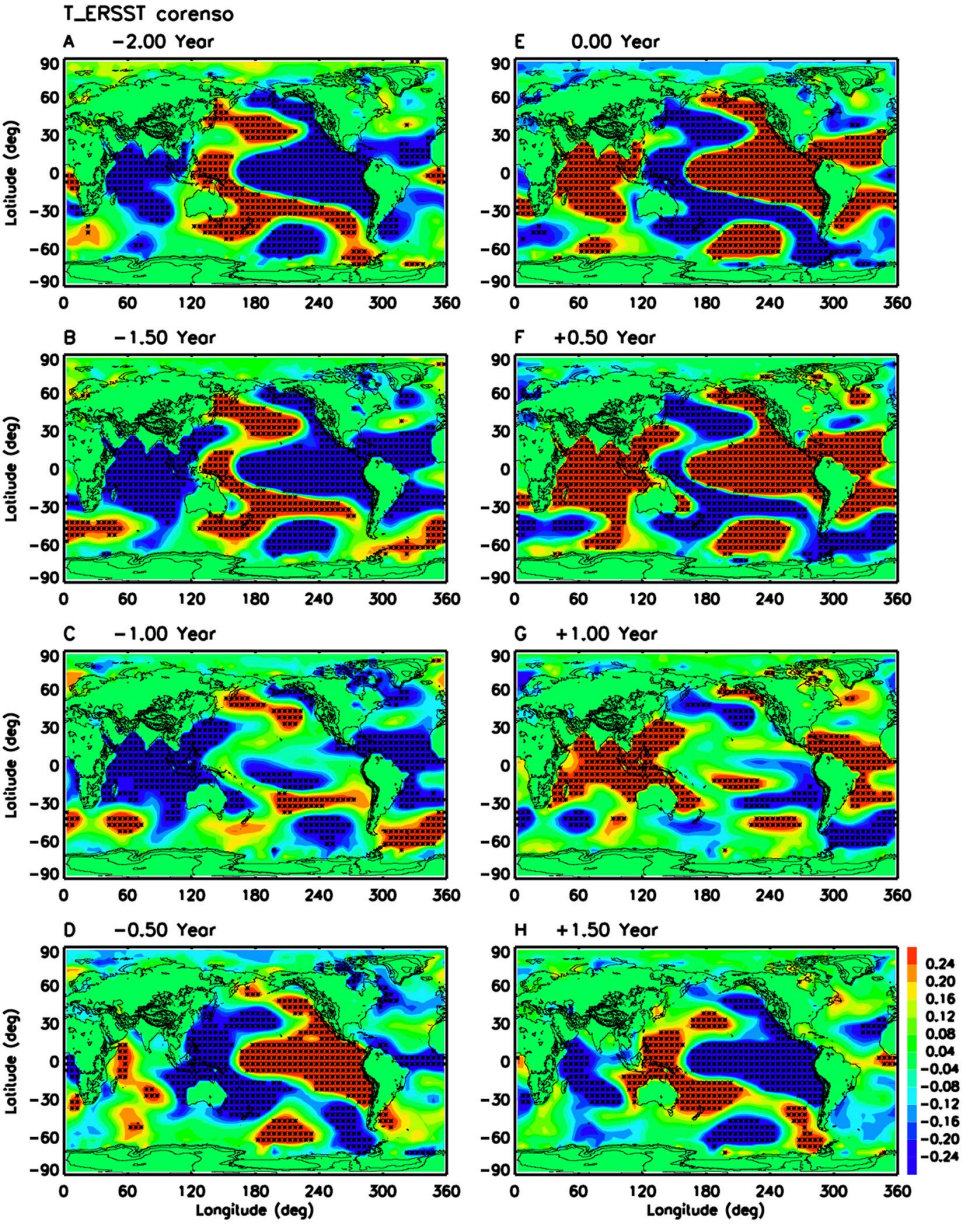
Evolution of global SST associated with ENSO lifecycle. Shadings show lag-correlation of 137 years (1880–2016) of global ERSST anomaly with Nino3.4 SST anomaly from (A) −2.0 years to (H) +1.5 years. Black stars denote the grids with lag-correlation above 95% confidence level.

Figure 1 illustrates the evolution of global SST with ENSO lifecycle using 137 years (1880–2016) of ERSST data. Similar results are obtained with COBE2 SST (Supplementary Figure 1) and HadISST (not shown). These are updates of the analysis of Lin and Qian (2019)^23^ using the old Kaplan SST data, which did not cover well the southern hemisphere high latitudes (poleward of 40 S). Figure 1 clearly shows the evolution of significant SST anomalies around the globe throughout the ENSO lifecycle. At lag 0 year (Fig. 1e), which is the peak of El Nino, the ERSST data shows better the global SST pattern with warming over tropical central/eastern Pacific and northeast Pacific, but a “horseshoe” cooling pattern over western Pacific, northwest Pacific and southwest Pacific. In particular, the ERSST data shows a wave train pattern in Southern Ocean, which is missing in the old Kaplan SST data^23^. At lag −2 years (La Nina, Fig. 1a), the SST anomalies are simply revered in sign. When the ENSO lifecycle is at the transition phases from La Nina to El Nino (Fig. 1c) and from El Nino to La Nina (Fig. 1g), which are often called the “neutral phase”, significant SST anomalies still exist in Indian Ocean, Atlantic Ocean and extratropical Pacific Ocean. It is very important to note that the SST anomalies during the cold-to-warm transition (Fig. 1c) have opposite signs to those during warm-to-cold transition (Fig. 1g). Therefore, they should not be added together into a “neutral phase” composite as in many previous ENSO studies, because the significant anomalies with opposite signs will be cancelled out. Figure 1 suggests that we should use a new four-phase paradigm of ENSO (warm phase, cold phase, cold-to-warm transition, and warm-to-cold transition) to replace the traditional three-phase paradigm (warm phase, cold phase, and neutral phase).

Supplementary Fig. 2 shows a summary of the impacts of ENSO lifecycle on global SST. Different datasets demonstrate consistent significant anomalies over global oceans throughout ENSO lifecycle. Various mechanisms have been proposed for the global SST anomalies associated with ENSO, such as the “atmospheric bridge” mechanism^24–26^, and for persistence of SST anomaly into next year, such as the “re-emergence mechanism”^27,28^. Dynamically, the observed significant SST anomalies throughout ENSO lifecycle drive temperature, precipitation and atmospheric circulation anomalies over the globe^29–36^.

**Figure 2:**
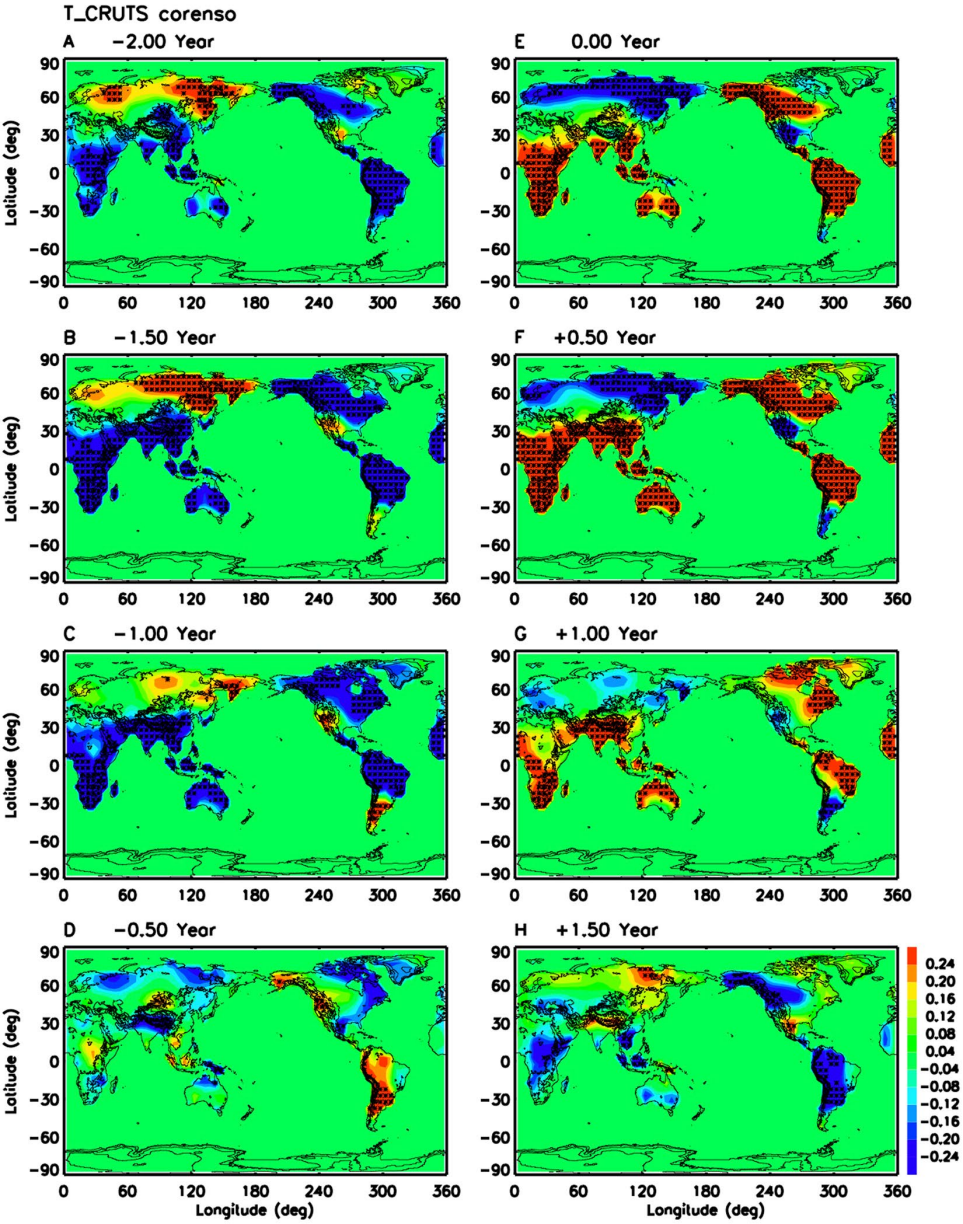
Same as Fig. 1 but for CRUTS land surface air temperature for 1901–2014.

Figure 2 demonstrates the impacts of ENSO lifecycle on land surface air temperature anomalies from 115 years (1901–2014) of CRUTS dataset. The correlation map during El Nino (Fig. 2e) can be compared with the current schematic for December-February (Trenberth *et al*.)^8^ since El Nino events tend to be phase-locked to northern winter^37,38^. The new findings from Fig. 2e are significant cold surface air temperature anomalies over Siberia and northern Europe, and warm temperature anomalies over Africa, India, Southeast Asia, West Australia, and part of South America. The correlation map for northern summer after El Nino (lag + 0.5 year, Fig. 2f) show similar new findings when compared to the current schematic for June-August (Trenberth *et al*.)^8^. During the transition phase from El Nino to La Nina (Fig. 2g), there are significant warm surface air temperature anomalies over west Africa, south Africa, south Asia, north Australia, northeast United States, and northeast Brazil, but cold temperature anomalies over Argentina. At lag + 1.5 years (Fig. 2h), cold temperature anomalies start to occupy South America, Alaska, western Canada and maritime continent. Evolution after La Nina (Fig. 2a–d) are simply reversed in sign. These results are generally confirmed by ERA-Interim reanalysis 2-meter temperature (Supplementary Fig. 3) and University of Delaware surface air temperature data (not shown). Overall, the summary plots (Supplementary Fig. 4) show significant impacts of ENSO lifecycle on land surface air temperature over all the continents, which are much wider than in the current schematic (Trenberth *et al*.)^8^. The corresponding maps of the linear regression coefficient between surface air temperature anomaly and Nino 3.4 SST anomaly (Supplementary Fig. 5) shows that the largest temperature responses are in NH high latitudes including Canada, Alaska and Russia.

**Figure 3:**
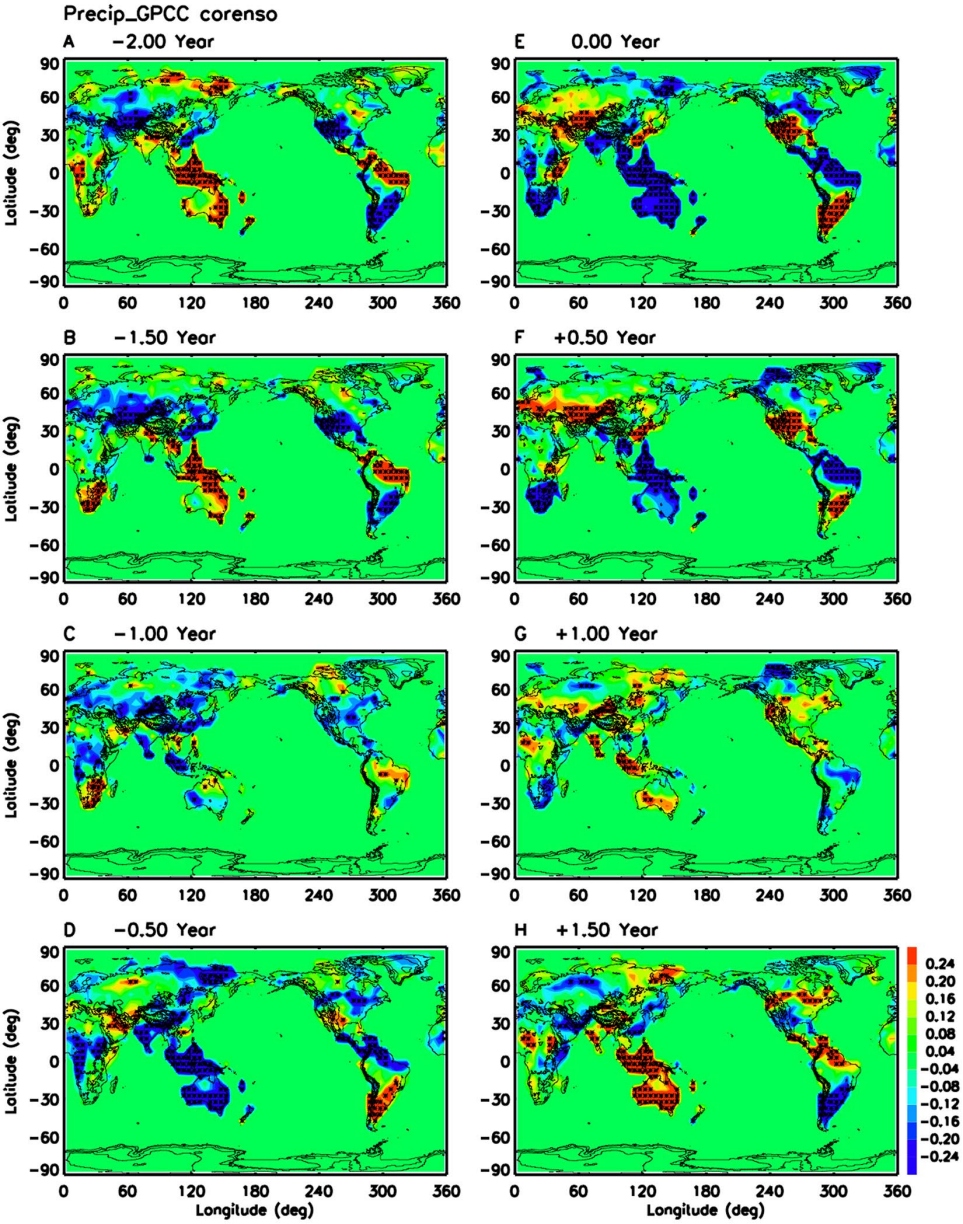
Same as Fig. 1 but for GPCC land surface precipitation for 1901–2013.

Figure 3 illustrates impacts of ENSO lifecycle on global land surface precipitation based on 114 years (1900–2013) of GPCC dataset. Comparison of El Nino phase (Fig. 3e) with current schematic (Trenberth *et al*.)^8^ reveals previously unknown strong wet anomalies over southeast China, central Asia and Middle East, and significant dry anomalies over west Africa, India, north Siberia, and central America. Comparison of northern summer after El Nino (lag + 0.5 year, Fig. 3f) with the current schematic (Trenberth *et al*.)^8^ shows newly found significant wet anomalies over central Asia, Middle East and Argentina, and strong dry anomalies over south Africa, southeast Asia and northern Canada. During the transition phase from El Nino to La Nina (Fig. 3g), significant wet anomalies start to develop over south India, maritime continent, west Africa and west Australia, while dry anomalies appear over Middle East. These anomalies grow much wider 6 months later (Fig. 3h). Over other continents such as north America and south America, strong ENSO impacts cover a larger area than what we knew before. For example, the significant ENSO impact on California droughts (Fig. 3a,b,e,f) is not well-depicted in the widely-used schematic. The results from GPCC dataset are generally supported by CRUTS dataset (Supplementary Fig. 6) and merged satellite-gauge GPCP dataset (Supplementary Fig. 7). The corresponding maps of the linear regression coefficient between surface precipitation anomaly and Nino 3.4 SST anomaly (Supplementary Fig. 8) shows that the largest precipitation responses are in the tropics and subtropics.

ENSO has a strong seasonality with the amplitude of Nino3.4 SST anomaly being strongest in November-December-January^37,38^. The surface air temperature anomalies in the summer after El Nino (Fig. 2f) are significantly different from those in the summer before El Nino (Fig. 2d). The precipitation anomalies in the summer after El Nino (Fig. 3f) also have important differences from those in the summer before El Nino (Fig. 3d). Studies using high Nino3.4 SST anomaly during summer tend to combine the two summers together. Our results suggest that it is important to separate the summer after El Nino from that before El Nino since they are at different stages of the ENSO lifecycle.

**Figure 4:**
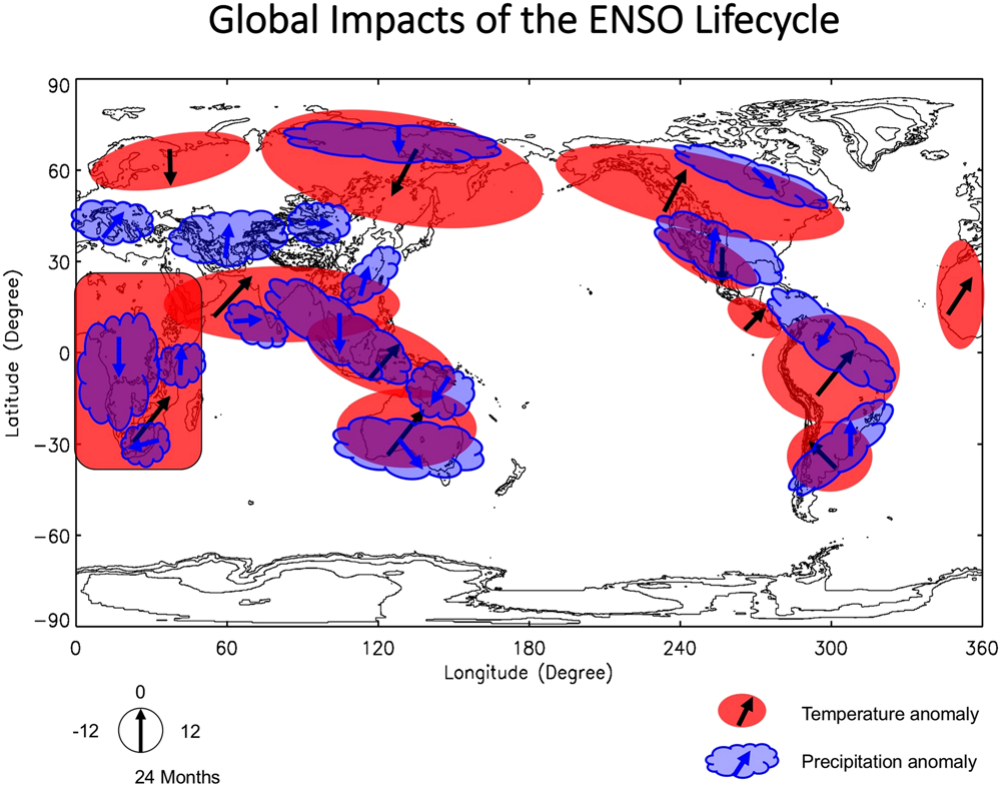
The new schematic of global impacts of ENSO during its whole lifecycle. Red shadings represent significant temperature anomalies, while blue clouds denote significant precipitation anomalies. Arrows denote the time lag of maximum anomalies with respect to Nino3.4 SST anomaly. Phase clock is shown under the schematic. The interval of background topography contours is 1000 m.

Figure 4 demonstrates our new schematic of global impacts of ENSO lifecycle on surface air temperature and precipitation. We only include the significant temperature and precipitation anomalies that are supported by at least two different datasets. Comparison of Fig. 4 with the current schematic (Trenberth *et al*.)^8^ shows that the impacts of ENSO are much wider than those shown in the widely-used schematic. The largest differences are in Europe, Asia and Africa. ENSO significantly modulates surface air temperature in northern Europe, north Asia, south Asia, and entire Africa, and precipitation in southern Europe, central Asia, northern north Asia, and central Africa. For other continents such as north America and south America, significant ENSO impacts cover a larger area than those shown in the widely-used schematic. It is important to note that the so-called “neutral phases” are associated with significant temperature and precipitation anomalies, and anomalies during cold-to-warm transition and warm-to-cold transition have opposite signs.

## Methods

Datasets used in this study are listed in Supplementary Table 1. The ENSO index used in this study is Nino3.4 SST from ERSST dataset. We have tested Nino3.4 SST from COBE2 SST and HadISST datasets and the results are similar. We follow the traditional methods for analysing ENSO’s global impacts^16–22^. Linear trend (a single trend calculated for all months together) and composite seasonal cycle are first removed from all datasets. To isolate the interannual ENSO signals from the decadal variability and higher-frequency variability, the anomalies are then filtered with a 3–6 year butterworth filter (Murakami)^39^. We also tested a wider 2–10 year filter and the results are similar. Lag-correlation is calculated with the Nino3.4 SST anomaly. Statistical significance is evaluated following Oort and Yienger^40^.

## Supplementary information

Supplementary Information

## Data Availability

Datasets used in this study are from NOAA ESRL/PSD Climate Data Archive https://www.esrl.noaa.gov/psd/ and NCAR Research Data Archive https://rda.ucar.edu.
