# Supplementary material for: A New Picture of the Global Impacts of El Nino-Southern Oscillation
Source: Sci Rep. 2019 Nov 26;9:17543. doi: 10.1038/s41598-019-54090-5 (PMC6879734; doi:10.1038/s41598-019-54090-5)
Supplement: Supplementary file 1 — Supplementary Information [file 41598_2019_54090_MOESM1_ESM.pdf]

A New Picture of the Global Impacts of El Nino-Southern Oscillation: Supplementary information

Jialin Lin[1,*] and Taotao Qian[1]

[1]Atmospheric Science Program, The Ohio State University, U.S.A.
**Corresponding author (email: lin.789@osu.edu)*

Contents of this file

Supplementary Table 1

Supplementary Figures 1-8

References 41-52

Supplementary Table 1. Datasets used in this study

VARIABLES	DATASETS	TIME PERIOD	REFERENCE
**Sea Surface Temperature**	Extended Reconstructed Sea Surface Temperature (ERSST) v4	1880-2016	Huang et al. (2015)[41]
	Hadley Centre Sea Ice and Sea Surface Temperature (HadISST1)	1880-2016	Rayner et al. (2003)[42]
	Centennial In Situ Observation-Based Estimates (COBE2)	1880-2016	Hirahara et al. (2014)[43]
**Land Surface Air Temperature**	Climatic Research Unit Surface Air Temperature (CRUTS)	1901-2014	Harris et al. (2014)[44]
	University of Delaware Surface Air Temperature (UDEL)	1900-2014	Willmott and Robeson (1995)[50]
	European Centre for Medium-Range Weather Forecasts (ECMWF) ERA-Interim Reanalysis	1979-2016	Dee et al. (2011)[47]
**Surface Precipitation**	Global Precipitation Climatology Centre (GPCC)	1901-2013	Schneider et al (2017)[51]
	Climatic Research Unit Precipitation (CRUTS)	1901-2014	Harris et al. (2014)[44]
	Global Precipitation Climatology Project (GPCP)	1979-2016	Adler et al. (2003)[52]
**Sea Level Pressure and Upper Level Geopotential Height**	National Oceanic and Atmospheric Administration (NOAA) 20Cv2 Reanalysis	1880-2012	Compo et al. (2011)[45]
	National Center for Environmental Prediction (NCEP) Reanalysis	1948-2016	Kalnay et al. (1996)[46]
	European Centre for Medium-Range Weather Forecasts (ECMWF) ERA-Interim Reanalysis	1979-2016	Dee et al. (2011)[47]

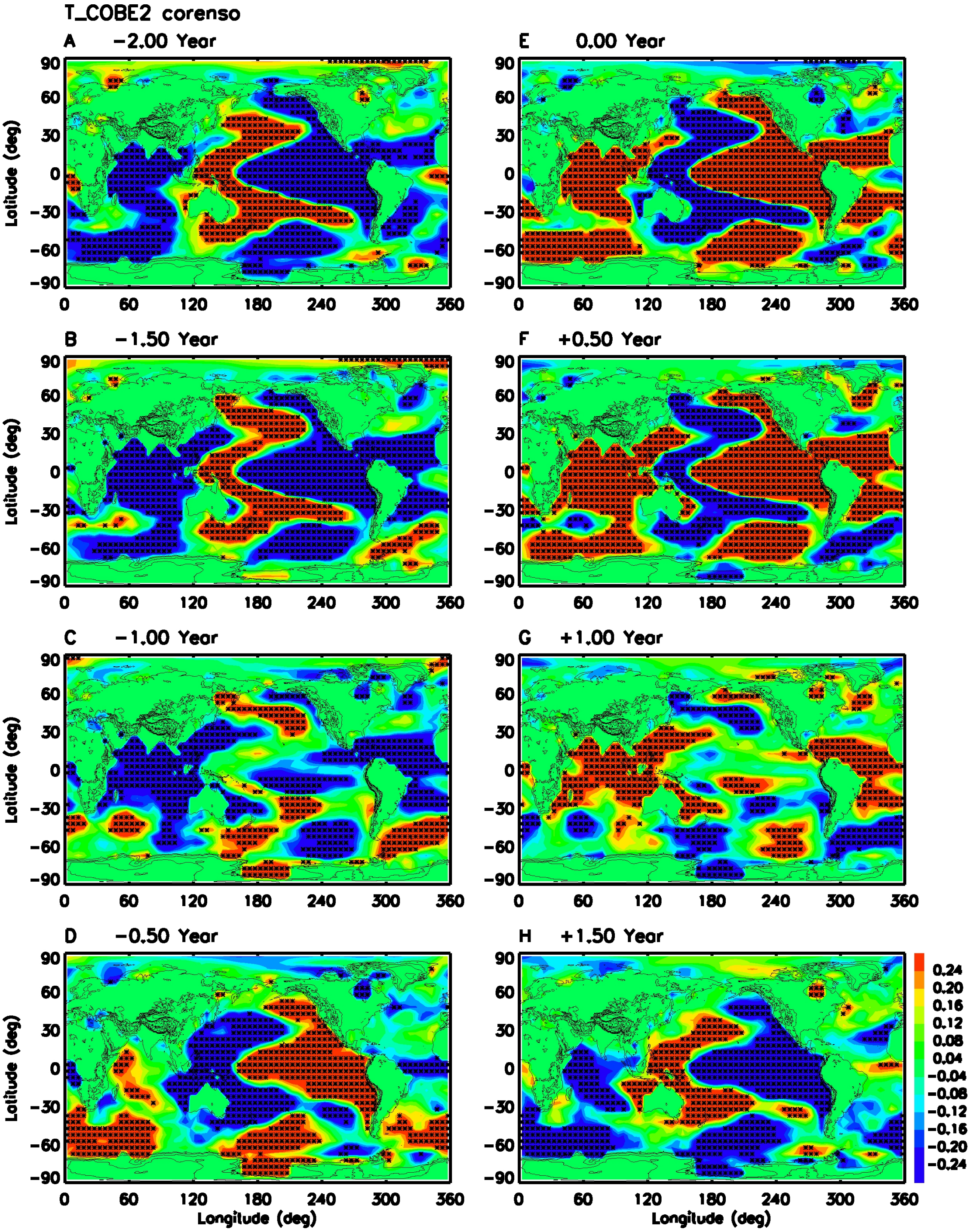

Supplementary Figure 1. Same as Figure 1 but for COBE2 SST for 1880-2016.

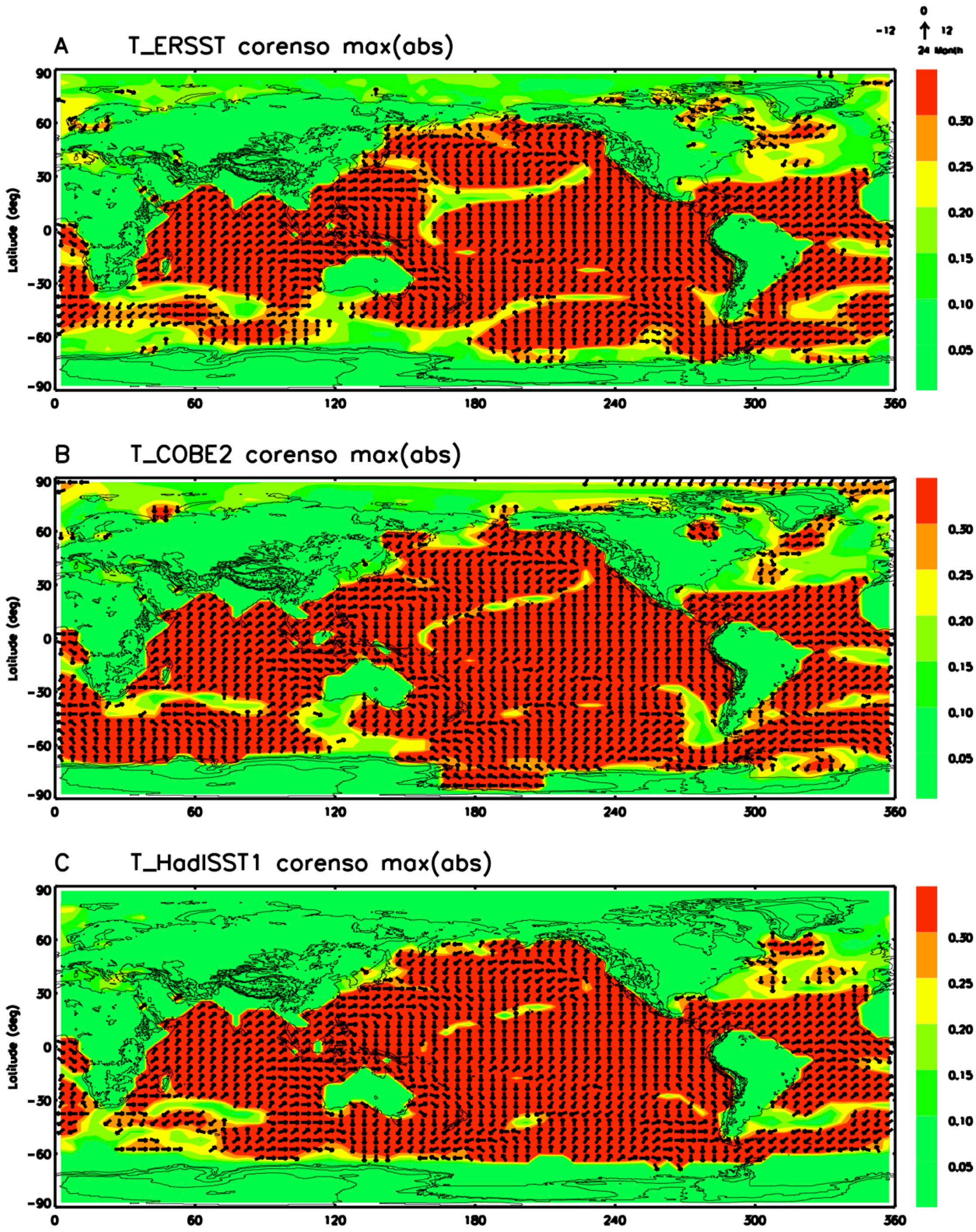

Supplementary Figure 2. Summary of impact of ENSO lifecycle on global SST for different datasets. (A) ERSST, (B) COBE2, and (C) HadISST1. Shadings show the maximum lag-correlation with Nino3.4 SST anomaly at each grid. Arrows denote the grids with maximum lag-correlation above 95% confidence level, and arrow directions represent the time lag of maximum correlation with respect to Nino3.4 SST anomaly. Phase clock is shown on upper-right corner.

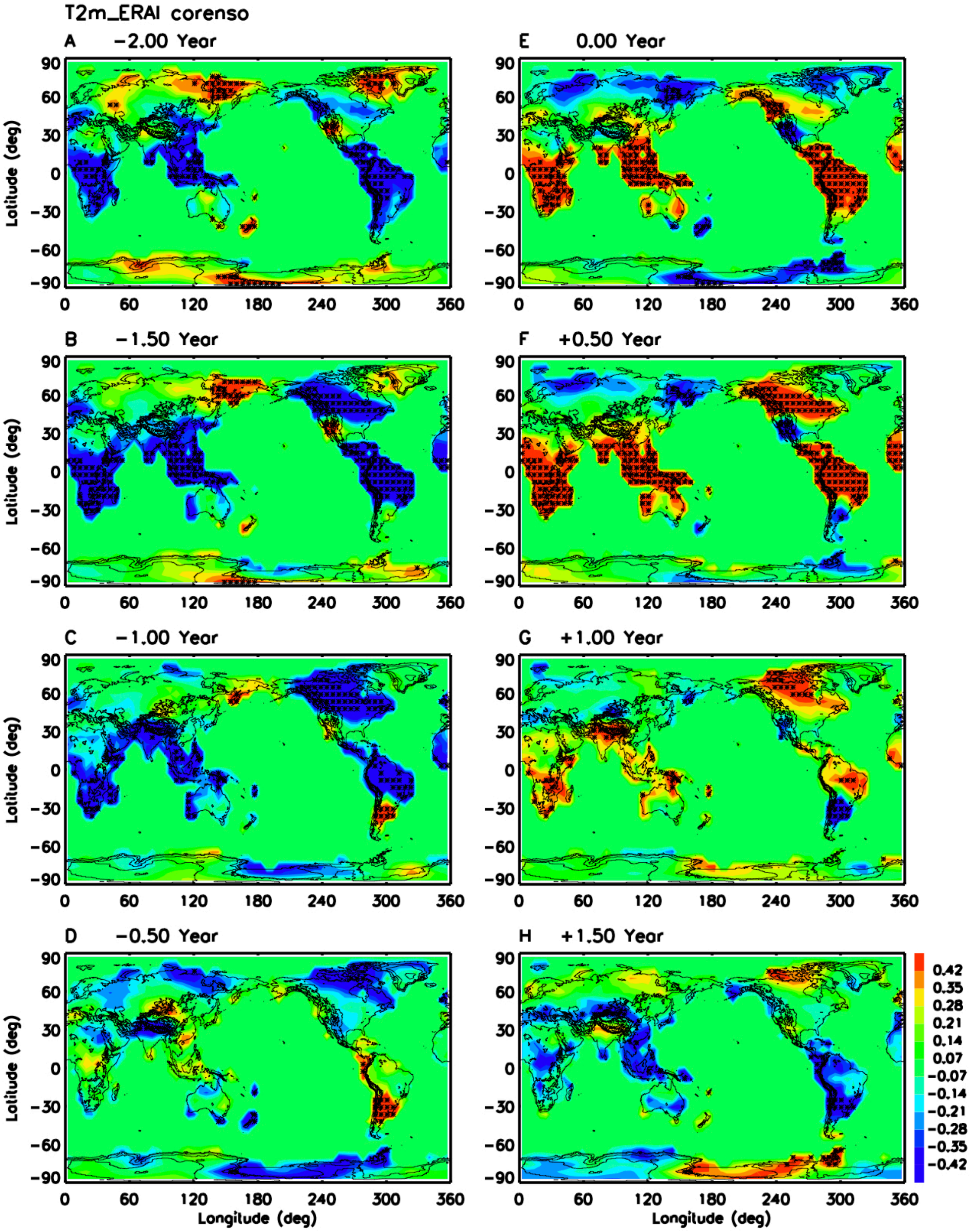

Supplementary Figure 3. Same as Figure 2 but for ERA-Interim 2m temperature for 1979-2016.

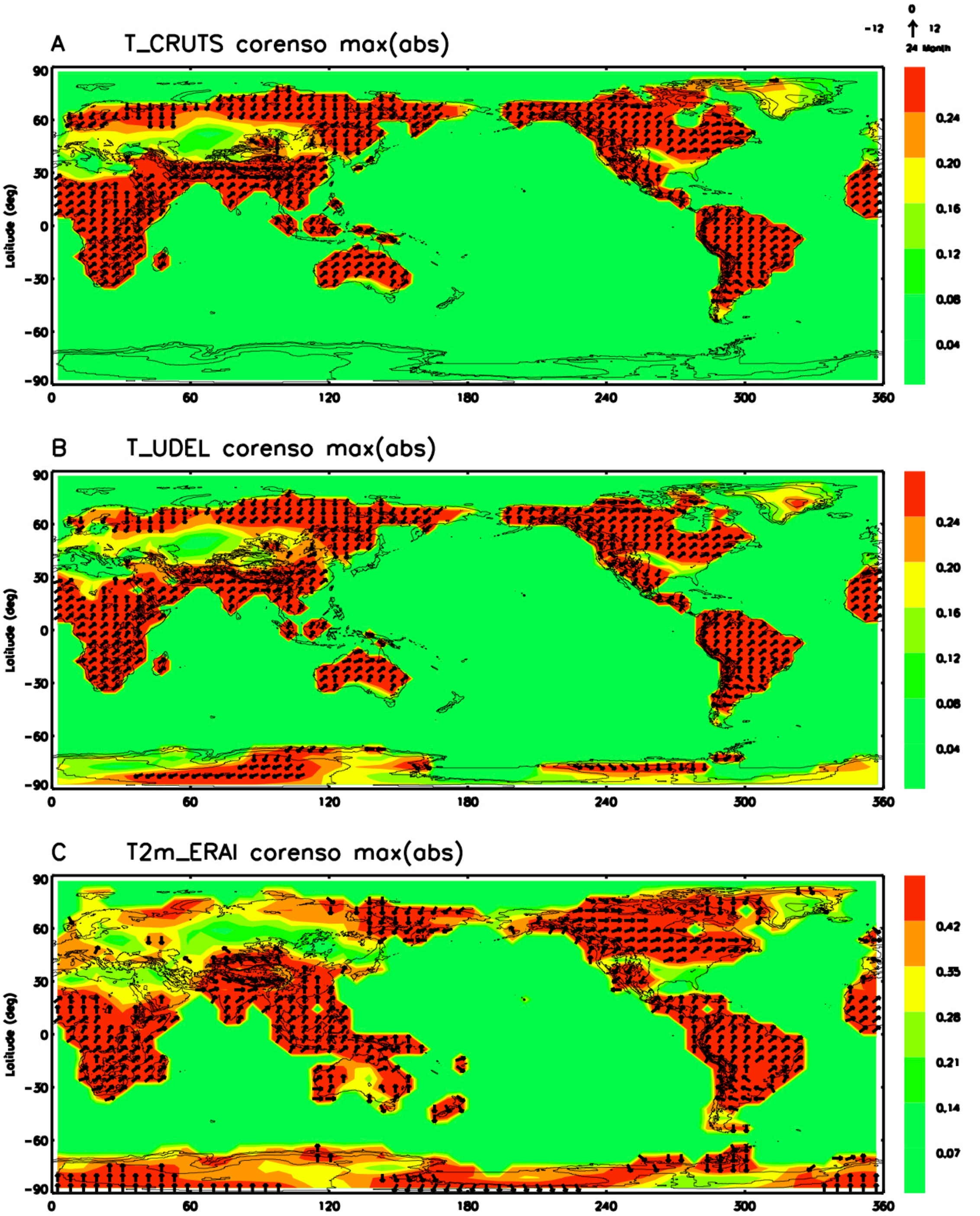

Supplementary Figure 4. Same as Supplementary Figure 2 but for global land surface air temperature for different datasets. (A) CRUTS, (B) University of Delaware, and (C) ERA-Interim reanalysis.

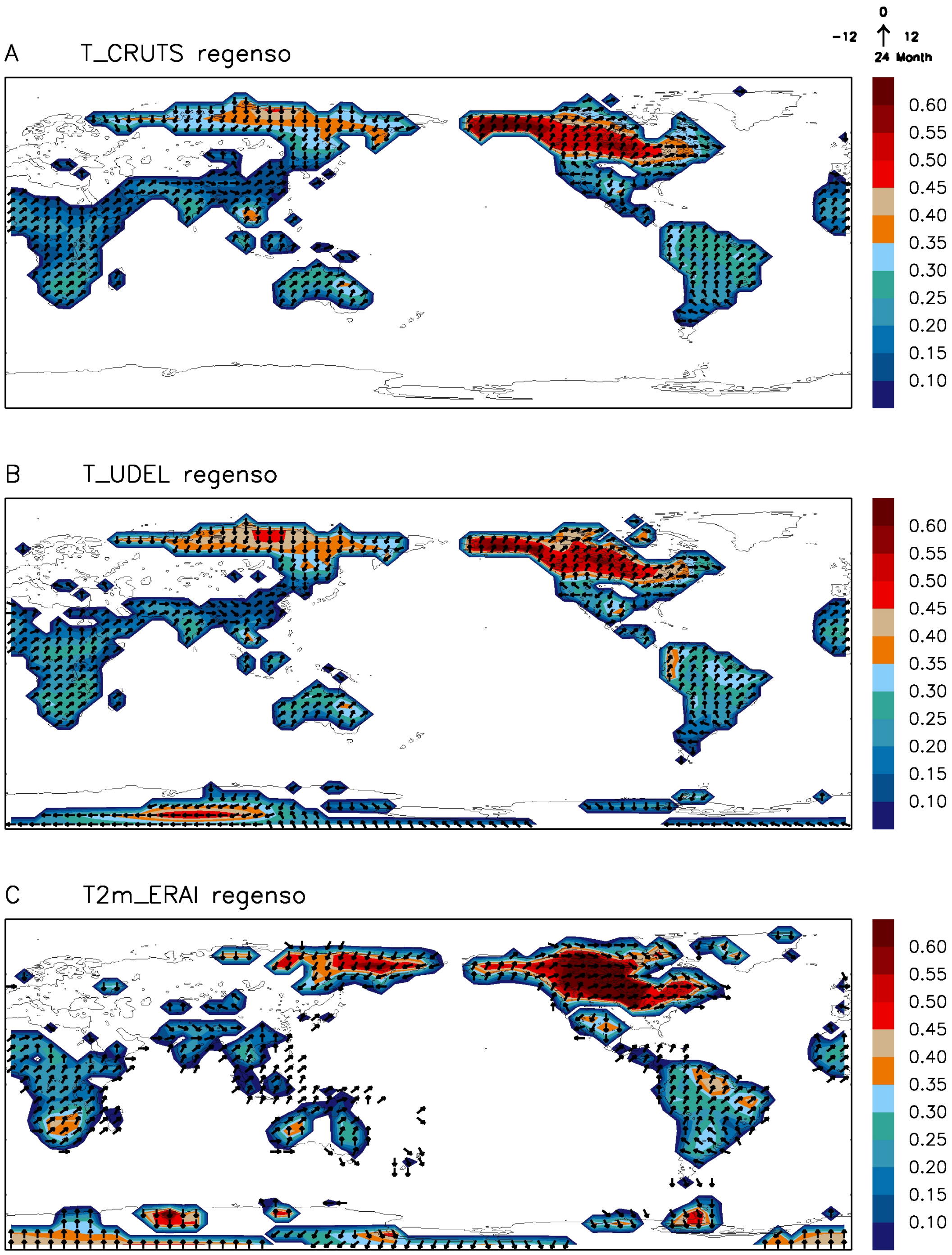

Supplementary Figure 5. Same as Supplementary Figure 4 but for the maximum lag-regression coefficient between land surface air temperature anomaly and Nino3.4 SST anomaly (unit: $^{o}C/^{o}C$).

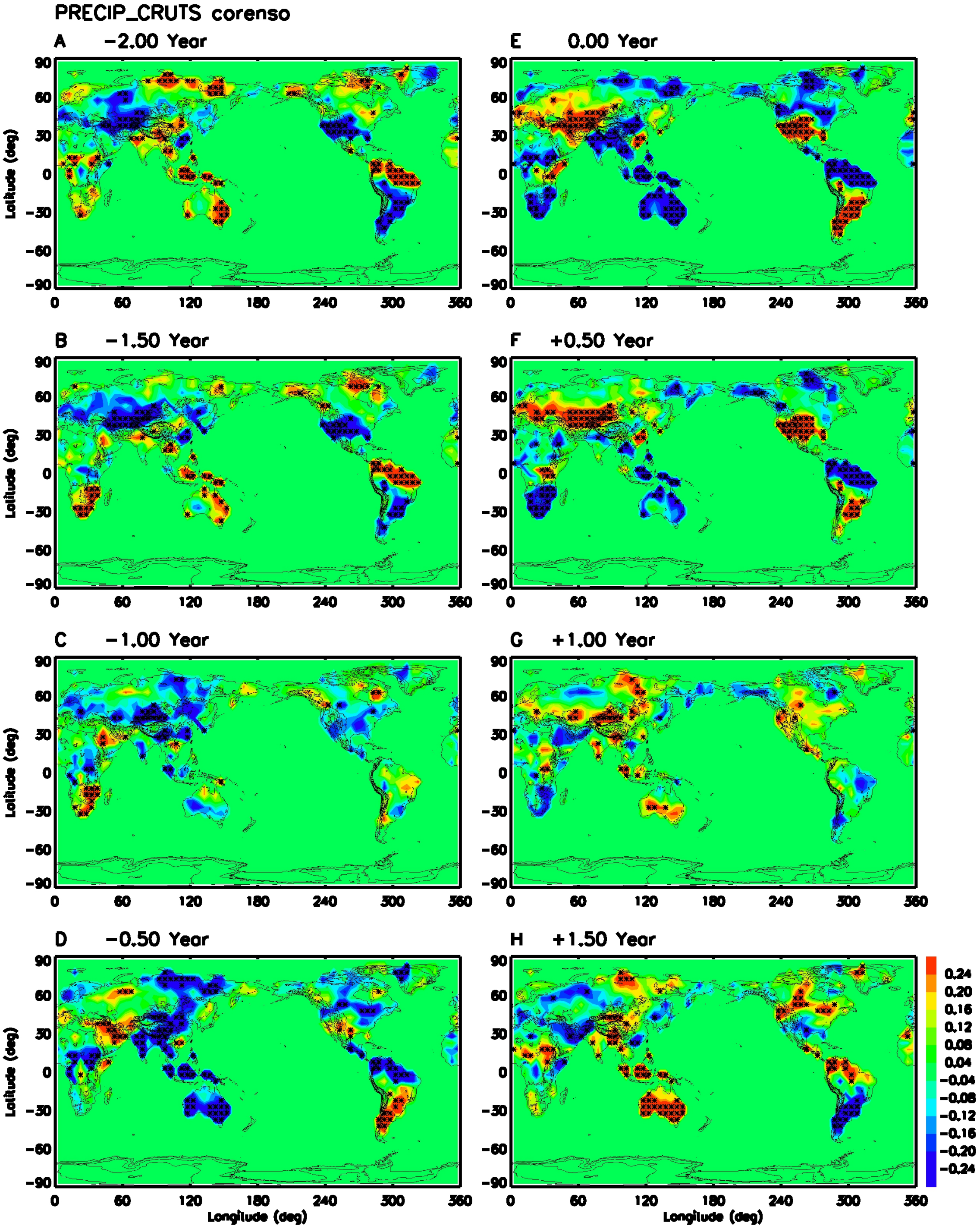

Supplementary Figure 6. Same as Figure 3 but for CRUTS precipitation for 1901-2016.

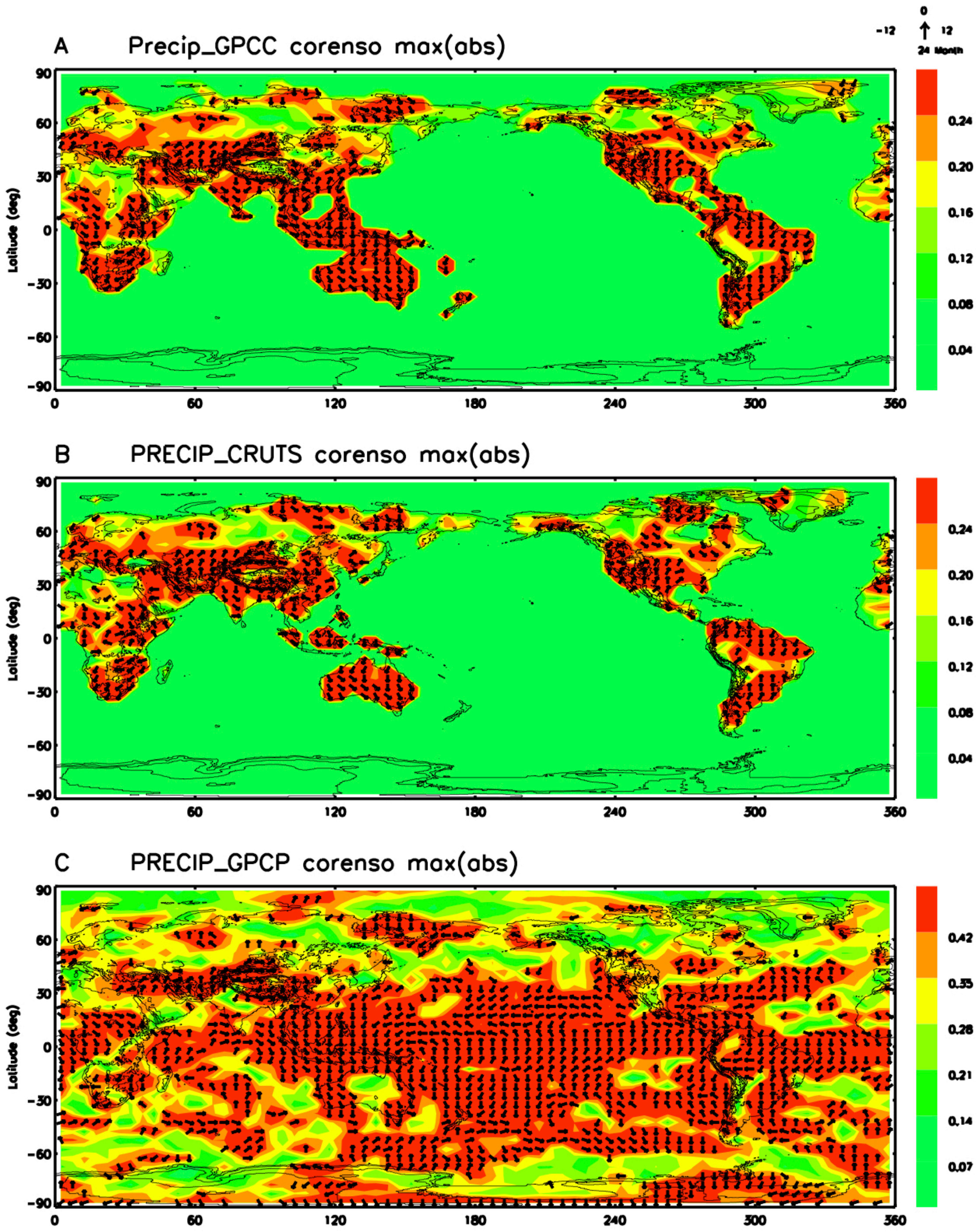

Supplementary Figure 7. Same as Supplementary Figure 2 but for global precipitation for different datasets. (A) GPCC, (B) CRUTS, and (C) GPCP.

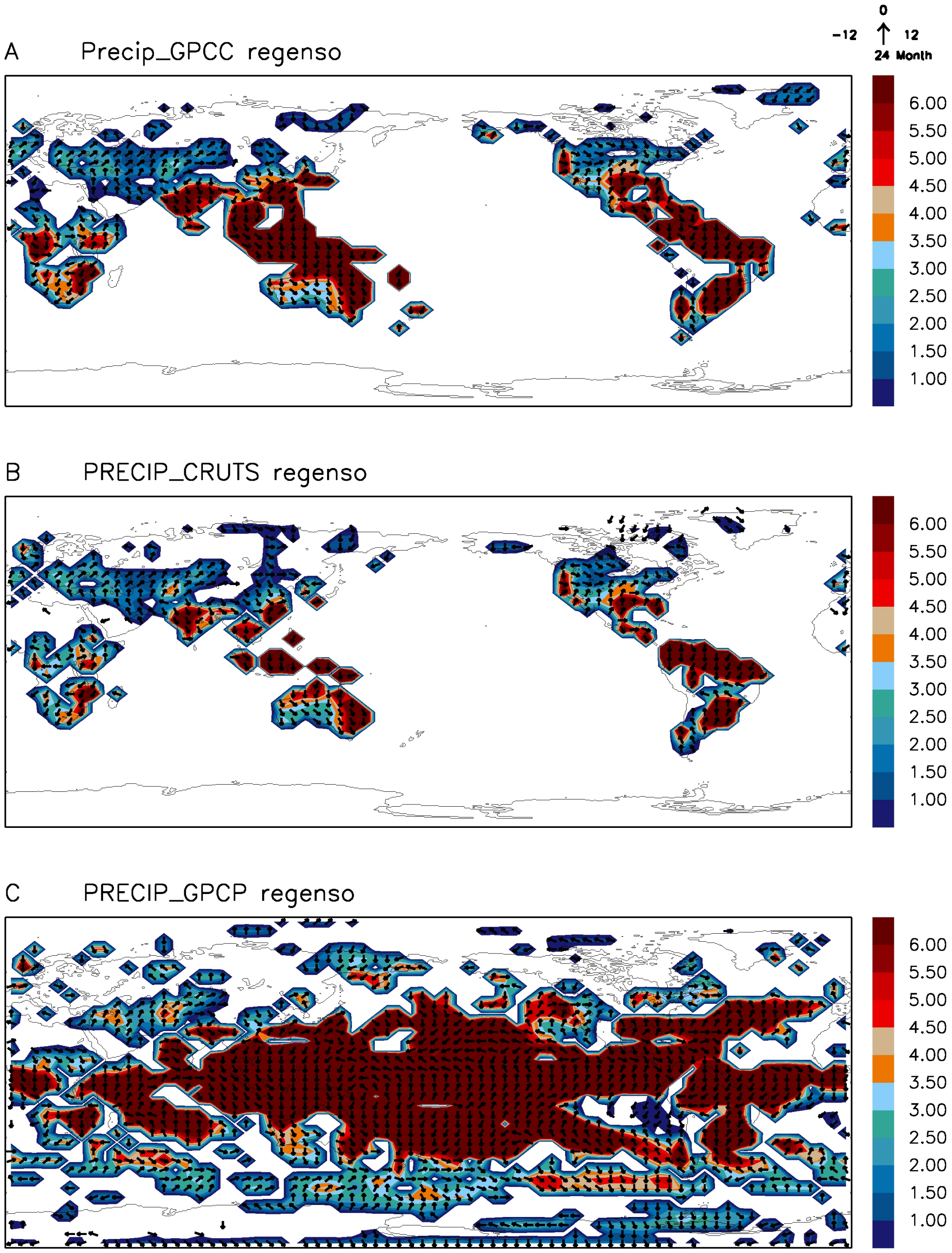

Supplementary Figure 8. Same as Supplementary Figure 7 but for the maximum lag-regression coefficient between land surface precipitation anomaly and Nino3.4 SST anomaly (unit: mm/month/^{o}C).

References

41. Huang, B., P. Thorne, T. Smith, W. Liu, J. Lawrimore, V. Banzon, H. Zhang, T. Peterson, and M. Menne, Further Exploring and Quantifying Uncertainties for Extended Reconstructed Sea Surface Temperature (ERSST) Version 4 (v4). Journal of Climate, 29, 3119-3142.(2015)

42. Rayner, N. A.; Parker, D. E.; Horton, E. B.; Folland, C. K.; Alexander, L. V.; Rowell, D. P.; Kent, E. C.; Kaplan, A., Global analyses of sea surface temperature, sea ice, and night marine air temperature since the late nineteenth century J. Geophys. Res.Vol. 108, No. D14, 4407 10.1029/2002JD002670.(2003)

43. Hirahara, S., Ishii, M., and Y. Fukuda, Centennial-scale sea surface temperature analysis and its uncertainty. J of Climate, 27, 57-75.(2014)

44. Harris et al. Updated high-resolution grids of monthly climatic observations - the CRU TS3.10 Dataset. https://crudata.uea.ac.uk/cru/data/hrg/(2014)

45. Compo, G.P., et al. 2011: The Twentieth Century Reanalysis Project. Quarterly J. Roy. Meteorol. Soc., 137, 1-28.

46. Kalnay et al., The NCEP/NCAR 40-year reanalysis project, Bull. Amer. Meteor. Soc., 77, 437-470.(1996)

47. Dee, D. P., et al. The ERA-Interim reanalysis: configuration and performance of the data assimilation system, Q. J. Roy. Meteorol. Soc., 137, 553-597.(2011)

48. Hansen, J., R. Ruedy, M. Sato, and K. Lo, 2010: Global surface temperature change. *Rev. Geophys.*, 48, RG4004, doi:10.1029/2010RG000345.

49. Harris, I., Jones, P. D., Osborn, T. J., and Lister, D. H., Updated high-resolution grids of monthly climatic observations - the CRU TS3.10 Dataset, Int. J. Climatol., 34, 623-642.(2014)

50. Willmott, C. J. and S. M. Robeson, Climatologically Aided Interpolation (CAI) of Terrestrial Air Temperature. International Journal of Climatology, 15, 221-229.(1995)

51. Schneider et al, Evaluating the Hydrological Cycle over Land Using the Newly-Corrected Precipitation Climatology from the Global Precipitation Climatology Centre (GPCC). Atmosphere 8(3), 52(2017)

52. Adler, R. F., G. J. Huffman, A. Chang, R. Ferraro, P. Xie, J. Janowiak, B. Rudolf, U. Schneider, S. Curtis, D. Bolvin, A. Gruber, J. Susskind, P. Arkin, and E. Nelkin, The version 2 Global Precipitation Climatology Project (GPCP) monthly precipitation analysis (1979-present). J. Hydrometeor, 4(6), 1147-1167.(2003)
